# An Immunoregulatory Role for Complement Receptors in Murine Models of Breast Cancer

**DOI:** 10.3390/antib10010002

**Published:** 2021-01-08

**Authors:** Fazrena Nadia Md Akhir, Mohd Hezmee Mohd Noor, Keith Weng Kit Leong, Jamileh A. Nabizadeh, Helga D. Manthey, Stefan E. Sonderegger, Jenny Nga Ting Fung, Crystal E. McGirr, Ian A. Shiels, Paul C. Mills, Trent M. Woodruff, Barbara E. Rolfe

**Affiliations:** 1 Australian Institute for Bioengineering and Nanotechnology, The University of Queensland, St Lucia, QLD 4072, Australia; fazrena@utm.my (F.N.M.A.); Keith_Leong@immunol.a-star.edu.sg (K.W.K.L.); j.nabizadeh@uq.edu.au (J.A.N.); h.manthey@uq.edu.au (H.D.M.); s.sonderegger@uq.edu.au (S.E.S.); j.fung1@uq.edu.au (J.N.T.F.); c.mcgirr@uq.edu.au (C.E.M.); 2 School of Biomedical Sciences, The University of Queensland, St Lucia, QLD 4072, Australia; t.woodruff@uq.edu.au; 3 School of Veterinary Science, The University of Queensland, Gatton, QLD 4343, Australia; hezmee@upm.edu.my (M.H.M.N.); ian.shiels@uq.edu.au (I.A.S.); p.mills@uq.edu.au (P.C.M.)

**Keywords:** complement C5a, complement C3a, complement receptors, mammary carcinoma, immunoregulation, tumor infiltrating leukocytes

## Abstract

The complement system has demonstrated roles in regulating tumor growth, although these may differ between tumor types. The current study used two murine breast cancer models (EMT6 and 4T1) to investigate whether pharmacological targeting of receptors for complement proteins C3a (C3aR) and C5a (C5aR1) is protective in murine breast cancer models. In contrast to prior studies in other tumor models, treatment with the selective C5aR1 antagonist PMX53 had no effect on tumor growth. However, treatment of mice with a dual C3aR/C5aR1 agonist (YSFKPMPLaR) significantly slowed mammary tumor development and progression. Examination of receptor expression by quantitative polymerase chain reaction (qPCR) analysis showed very low levels of mRNA expression for either *C3aR* or *C5aR1* by EMT6 or 4T1 mammary carcinoma cell lines compared with the J774 macrophage line or bone marrow-derived macrophages. Moreover, flow cytometric analysis found no evidence of C3aR or C5aR1 protein expression by either EMT6 or 4T1 cells, leading us to hypothesize that the tumor inhibitory effects of the dual agonist are indirect, possibly via regulation of the anti-tumor immune response. This hypothesis was supported by flow cytometric analysis of tumor infiltrating leukocyte populations, which demonstrated a significant increase in T lymphocytes in mice treated with the C3aR/C5aR1 agonist. These results support an immunoregulatory role for complement receptors in primary murine mammary carcinoma models. They also suggest that complement activation peptides can influence the anti-tumor response in different ways depending on the cancer type, the host immune response to the tumor and levels of endogenous complement activation within the tumor microenvironment.

## 1. Introduction

Breast cancer is the most common cancer diagnosed in women world-wide, accounting for 25% of all cancers and 15% of cancer deaths in women [1]. Despite significant advances in understanding the underlying biology, some forms of disease remain resistant to current treatments—in particular, triple-negative breast cancers [2]. Chronic inflammation is linked to the development and progression of many cancers [3,4]. As powerful mediators of inflammation, complement proteins have been implicated for a role in tumorigenesis [5], with elevated complement regulatory proteins and activation fragments identified as biomarkers and prognostic indicators for many cancers, including breast cancer [6,7,8].

Comprising more than 40 plasma- and membrane-bound proteins, the overall function of the complement system is to regulate inflammation, facilitate immune defense mechanisms and maintain tissue homeostasis [9]. The complement system has long been recognized to contribute to anti-tumor defense mechanisms via complement-dependent cytotoxicity (CDC) [10] and antibody-dependent cell-mediated cytotoxicity (ADCC) [11]. The upregulation of complement inhibitory proteins is thought to be an important mechanism by which cancer cells evade complement-mediated destruction [12,13,14]. There is also increasing evidence of a role for the complement activation products C3a and C5a in regulating tumor growth and metastasis. C3a and C5a are small polypeptides with 36% homology [15]. C5a is one of the most potent inflammatory proteins and chemoattractant for neutrophils, monocytes and macrophages [16]. It binds two specific receptors, C5a receptor (CD88/C5aR1) and C5a receptor-like 2 (C5L2/C5aR2), of which the former (G-protein coupled C5aR1) is thought to be the predominant driver of biological activity [17,18]. C3a binds a single receptor, C3aR1, which like C5aR1 is a G-protein-coupled receptor expressed primarily by cells of myeloid origin [15]. Originally thought to have similar pro-inflammatory effects to C5a, C3a is now known to exert a range of apparently contradictory immunomodulatory functions—attenuating neutrophil mobilization in response to injury [19] but inducing production of pro-inflammatory cytokines [20].

Kim and co-workers [21] were the first to show a direct role for complement C5a in regulating breast cancer growth, demonstrating that over-expression by EMT6 mammary tumor cells protected against tumor growth in mice. Tumor inhibitory effects of complement proteins were confirmed by Bandini et al., who showed in an autochthonous mammary carcinoma model that Her2/neu-driven carcinogenesis is accelerated in C3-deficient mice [22]. Conversely, Vadrevu and co-workers found no effect of C5aR1 deficiency on primary tumor growth, but metastatic tumor burden was reduced in the murine 4T1 breast cancer model [23]. Moreover, C5a-C5aR1 signaling has been associated with cancer progression and poor prognosis in breast cancer patients [8,23]. Tumor-promoting effects of C5a have also been reported in other murine cancer models, including cervical [24], lung [25], ovarian [26] and melanoma [27]. Less is known about the role of C3a in tumor growth, although our laboratory and others have demonstrated that inhibition of C3aR signaling inhibits the growth of murine melanoma, colon, breast [28], intestinal [29] and lung cancer [30]. Whilst most of these studies have identified indirect (immune-mediated) mechanisms responsible for regulating tumor growth, tumor-intrinsic effects have also been reported [21,31]. Other effects are also possible. For example, there is evidence that C5a promotes vascularization of ovarian tumors [26], while C3a has been demonstrated to promote leptomeningeal metastasis by disrupting the blood-cerebrospinal fluid barrier [32].

To further explore the effects of the complement receptors in mammary cancer, we used two syngeneic murine mammary carcinoma models: EMT6, which is weakly estrogen receptor (ER)-positive [33], and 4T1, a model of triple-negative breast cancer [34]. Mice were treated with a selective C5aR1 antagonist (AcF-[OPdChaWR]; PMX53) [35], which has been demonstrated to effectively reduce C5a-mediated inflammatory responses in animal disease models [36,37,38], including murine melanoma [27], cervical [24] and lung [25] cancers. Because there are no selective agonists for mouse C5aR1 suitable for in vivo application, and native C3a and C5a proteins are highly susceptible to proteolysis by serum carboxypeptidases [39], mice were treated with a dual C3aR/C5aR1 agonist (YSFKPMPLaR; EP54) [40]. Although we found no effect of C5aR1 inhibition on tumor growth, treatment with the dual C3aR/C5aR1 agonist inhibited tumor development and progression. The results suggest that the anti-tumor effects are indirect, possibly due to enhancement of the T lymphocyte-mediated anti-tumor response.

## 2. Materials and Methods

### 2.1. Drugs

The dual C3aR/C5aR1 agonist YSFKPMPLaR (EP54) and the cyclic peptide C5aR1 antagonist AcF-[OP(D-Cha)WR] (PMX53) were synthesized in-house using previously described methods [41,42,43]. Drugs for injection were diluted in either 5% glucose or 0.9% saline. 

### 2.2. Cell Culture

Tumorigenic mouse mammary carcinoma EMT6 (ATCC *CRL-2755*) and 4T1 (ATCC *CRL-2539*) as well as J774A.1 macrophage (ATCC TIB-67) cell lines were obtained from the American Type Cell Culture Collection. EMT6 cells were maintained in Waymouths medium (Invitrogen, Carlsbad, CA, USA) containing 10% heat-inactivated fetal calf serum (FCS; Moregate, Brisbane, QLD, Australia). 4T1 and J774 cells were grown in RPMI 1640 medium (Invitrogen) + 10% FCS. 

Bone marrow-derived macrophages (BMDM) were prepared by flushing femurs of 8- to 12-week-old mice with phosphate buffered saline (PBS). Bone marrow cells were seeded onto untreated culture plates and cultured for 7 days in RPMI + 10% FCS containing mouse colony stimulating factor (mCSF)-1 (50 ng/mL; BioLegend, San Diego, CA, USA). All cells were maintained at 37 °C in an atmosphere of 5% CO_2_ in air (Invitrogen).

### 2.3. Animals

Female BALB/c mice (Monash Animal Services, Melbourne, Aust), 6–8 weeks of age, were housed 4/cage in the UQBR animal facility, University of Queensland, with lighting schedules of a 12 h light/dark cycle, and water and standard rodent diet provided ad libitum. All procedures were approved by the University of Queensland Animal Ethics Committee Guidelines and conformed to the *Australian Code of Practice for the Care and Use of Animals for Scientific Purposes* (8th Edition, 2013).

#### Tumor Cell Injections and Drug Treatments

BALB/c mice (bodyweight approximately 20–25 g; n = 7–8 animals/group) were lightly anesthetized with isofluorane (1.5% in oxygen) and the left mammary fat pad injected with 0.5 × 10^6^ of either EMT6 or 4T1 cells in a total volume of 0.05 mL serum-free medium. Mice commenced daily sub-cutaneous (s.c.) injections with EP54 (1 or 3 mg/kg bodyweight), PMX53 (1 mg/kg bodyweight) or vehicle only (5% glucose or 0.9% saline solution), either from the time of tumor injection (day 0) or once tumors became palpable (approximately day 7). These drug doses were previously shown to be effective in other mouse models of disease [43,44].

Mice were monitored daily and once tumors became palpable (at approximately day 7), tumor areas were measured daily by the same individual using digital Vernier calipers. Since it was not possible to measure tumor height accurately, and area measurements have been shown to correlate well with the mass of small tumors [45], tumor width and length were measured, and tumor areas calculated [46]. Once the largest tumor area had reached approximately 200 mm^2^, mice in all groups were euthanized and tumors removed from each mouse. Excised tumors were weighed, then processed for flow cytometric analysis. 

### 2.4. RNA Extraction and Quantitative Polymerase Chain Reaction (qPCR)

Total RNA was isolated from EMT6, 4T1 mammary carcinoma cells (n = 3), BMDM (n = 3) and J774 macrophages (n = 2) using the RNeasy plus Mini Kit (Qiagen, Hilden, Germany). RNA quality was determined and quantified by spectrophotometer (Nanodrop ND1000; Thermo Scientific, Waltham, MA, USA). Total RNA (1 μg) was then converted to cDNA using the iScript^™^ cDNA synthesis kit (Bio-Rad, Hercules, CA, USA). Taqman probes for *C3* (Mm01232779_m1), *Hc* (*C5*; Mm00439275_m1), *C3aR* (Mm02620006_s1) and *C5ar1* (Mm00500292_s1) (Applied Biosystems, Foster City, CA, USA) were used to amplify the target genes. Relative target gene expression to reference gene hypoxanthine guanine phosphoribosyl transferase (*Hprt*; Mm03024075_m1) was determined using the formula: 2^−∆CT^, where ∆CT = (Ct _(Target gene)_ − Ct _(Hprt)_). 

### 2.5. Calcium Mobilization Assay

Cells were seeded at 3 × 10^4^ cells/well into 96-well, black-walled, clear-bottom plates (Nunc^™^, Thermo Fisher Scientific) and allowed to attach overnight. Cells were loaded with Fluo-4 dye (Invitrogen), then transferred to a FlexStation III microplate reader (Molecular Devices, San Jose, CA, USA) before addition of EP54 (5 × 10^−4^ to 5 × 10^−9^ mol/L), and changes in fluorescence (λex = 485 nm; λem = 525 nm) were measured at 3 s intervals.

### 2.6. Flow Cytometric Analysis 

EMT6, 4T1 and J774 cells were detached from culture dishes by trypsinization or aspiration with ice-cold Dulbecco’s phosphate buffered saline (D-PBS; Ca and Mg free). Single cell suspensions were prepared from excised tumors by mechanical disaggregation, followed by filtration through 70 µm nylon cell strainers, and resuspended (0.5–2.0 × 10^6^ cells/mL) in calcium- and magnesium-free PBS containing 0.1% bovine serum albumin and 0.1% sodium azide (PBA). Cells were dispensed into 96-well plates (0.5–2.0 × 10^5^ viable cells/well) and pre-incubated with anti-CD16/32 (2.4G2; BioLegend) for 15 min to block Fc receptors. Cultured cells were incubated with fluorescein isothiocyanate (FITC)-conjugated rat anti-mouse C3aR (14D4; Hycult, Uden, NL, USA) or control Ig for 1 h. Cells from excised tumors were incubated with fluorophore-conjugated rat monoclonal antibodies for mouse surface leukocyte markers: CD45, CD11b, F4/80, Gr-1, CD25, CD3, CD4 and CD8a (all from BioLegend). To identify regulatory T cells (Tregs), cells were surface-stained, then fixed and permeabilized (FoxP3 Fix/Perm kit; BioLegend) for intracellular staining with anti-Foxp3 (BioLegend). To identify Th1, Th2 and Th17 subsets, cells were stimulated with phorbol 12-myristate 13-acetate (PMA; 50 ng/mL; Sigma) and ionomycin (10^-6^ M; Sigma) in the presence of Brefeldin A (5 µg/mL; BioLegend) for 4 h at 37 °C, surface-stained with anti-CD3 and CD4, then fixed, permeabilized and stained with antibodies to intracellular cytokines interleukin (IL)-4, interferon (IFN)-γ or IL-17A (BioLegend). Cell viability was determine using DRAQ7 (Cell Signaling, Danvers, MA, USA). Cells were analyzed on an Accuri C6 or LSR Fortessa X-20 flow cytometer (BD Biosciences, Franklin Lakes, NJ, USA) followed by data analysis with FlowJo software (Tree Star, Inc., Ashland, OR, USA). Gating strategies are shown in Supplementary Figure S1.

### 2.7. Statistical Analysis

All experiments were performed a minimum of two times, and values expressed as mean ± standard deviation (SD). Tumor growth was analyzed by two-way analysis of variance (ANOVA). All other data were analyzed using unpaired Student’s *t*-test, or one-way ANOVA followed by Dunnett’s multiple comparison tests and Bonferroni post-test (GraphPad Software Inc., San Diego, CA, USA). A *p*-value of <0.05 was considered significant.

## 3. Results

### 3.1. Effects of Pharmacological Modulation of C3aR/C5aR1 Signaling on Mammary Carcinoma Growth in Mice

**Figure 1:**
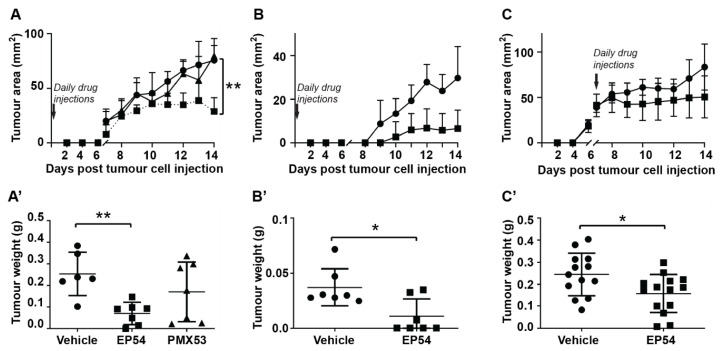
Effect of pharmacological modulation of C3aR/C5aR1 signaling on growth of murine mammary carcinomas. Tumor areas (mm^2^) (**A**–**C**) and excised tumor weights (g) at the end of trial (day 14) (**A’**–**C’**) in female BALB/c mice injected with (**A**,**A’**) EMT6 tumor cells and treated by daily sub-cutaneous (s.c.) injection with vehicle alone (5% dextrose, ●), dual C3aR/C5aR1 agonist (EP54; 1 mg/kg/day, ■) or C5a antagonist (PMX53; 1 mg/kg/day, ▲), commencing day 0. (**B**,**B’**) 4T1 tumor cells treated by daily s.c. injection with vehicle alone or EP54 (1 mg/kg/day), commencing day 0. (**C**,**C’**) Established EMT6 tumors treated by daily injection of EP54 from day 7 after tumor cell injection. Data expressed as mean ± standard deviation (SD). Results are representative of two separate experiments (*n* = 6–7/group); ** *p <* 0.01; * *p* < 0.05.

To determine the influence of C3aR/C5aR1 signaling on tumor development, mice were injected with EMT6 mammary carcinoma cells. On the same day (day 0), mice commenced daily injections with either C5aR1 antagonist, PMX53 (1 mg/kg/day), dual C3aR/C5aR1 agonist, EP54 (1 mg/kg/day) or vehicle (control). Tumors became palpable at approximately day 7 (Figure 1A). Caliper measurements showed that PMX53 had no significant effect on the growth of EMT6 tumors, but tumor growth was slowed by EP54 treatment (*p* < 0.01; Figure 1A). Excised tumor weight at day 14 was also significantly reduced in mice treated with EP54 (0.07 ± 0.05 g) compared with the control group (0.25 ± 0.1 g; *p* < 0.01; Figure 1A’). Health assessment scores showed that treatment with EP54 was associated with significantly less deterioration in general health of the mice and there was no significant change in body weight for any group: body weights for EP54-treated mice were 19.1 ± 1.6 g on day 1 and 19.2 ± 1.4 g at day 14 post-tumor induction, compared with 19.9 ± 1.7 g and 19.5 ± 2.2 g respectively, for the control (vehicle-treated) group. The reduction in tumor growth was not significantly enhanced by a higher dose of EP54 (3 mg/kg/day; data not shown), indicating that a dose of 1 mg/kg/day is sufficient. 

A similar trend was observed in mice injected with 4T1 tumors (Figure 1B,B’), with EP54 treatment slowing tumor growth (*p* < 0.01; Figure 1B). Excised tumor weights in the EP54-treated group (0.011 ± 0.016 g) were also significantly lower than the control group (0.037 ± 0.017 g; *p* < 0.05) (Figure 1B’). Having shown that EP54 treatment inhibits tumor initiation, we next investigated its effect on established EMT6 tumors. For these experiments, mice were injected with EMT6 cells, and once tumors were established (day 7), daily s.c. injections of EP54 (1 mg/kg/day) or saline alone (vehicle control) were commenced. As shown in Figure 1C,C’, tumor growth was significantly slowed (*p* < 0.01), and excised tumor weights were smaller in EP54-treated mice (0.16 ± 0.09 g) compared with the control group (0.24 ± 0.1 g; *p* < 0.05). For all experiments, there were no signs of drug toxicity, and mice showed no significant changes in bodyweight.

### 3.2. Expression of Complement Receptors C5aR1 (CD88) and C3aR by EMT6 and 4T1 Mammary Carcinoma Cell Lines

**Figure 2:**
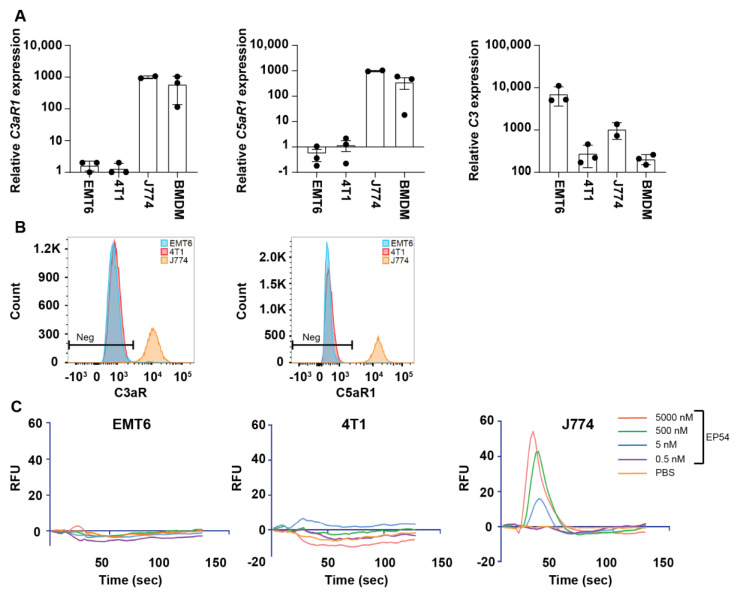
Complement receptor expression by mouse mammary carcinoma cell lines. (**A**) Quantitative polymerase chain reaction (qPCR) analysis shows relative expression of mRNA for *C3aR1*, *C5aR1* and *C3* by cultured EMT6 (n = 3) and 4T1 (n = 3) mammary carcinoma cell lines compared to J774 macrophages (n = 2) and bone marrow-derived macrophages (BMDM; n = 3). Data are normalized to the reference gene, *Hprt,* and expression is shown relative to J774 cells (mean ± SD). (**B**) Flow cytometric analysis shows C3aR and C5aR1 expression by EMT6 and 4T1 cell lines compared with J774 macrophages. (**C**) Dose-response to dual C3aR/C5aR1 agonist EP54 and phosphate buffered saline (PBS; negative control) for EMT6 and 4T1 mouse mammary carcinoma cells and J774 macrophages (positive control). Intracellular calcium levels were measured in real time using Fluo-4 dye, with change in relative fluorescence units (RFU) indicative of intracellular calcium flux. RFU was measured for 160 s with drug addition at 15 s. Results are representative of data collected from three separate experiments.

Having established that C3a/C5aR1 agonism is effective in inhibiting mammary tumor growth in mice, we next explored possible mechanisms responsible for the anti-tumor effects. Since Kim and co-workers [21] suggested that C5a may act directly on tumor cells, we first investigated receptor expression by cultured EMT6 and 4T1 cells. Analysis by qPCR showed that both cell lines expressed mRNA for *C3aR* and *C5aR1,* but at levels 600–750-fold lower than J774 cells and 300–500 times lower than BMDMs. Neither cell line expressed *Hc* (*C5*)*,* but *C3* expression by EMT6 cells was more than 7-fold higher than J774 macrophages and 30-fold higher than BMDM (Figure 2A). Flow cytometric analysis found no detectable expression of C3aR or C5aR1 protein by either tumor cell line, whereas J774 macrophages and BMDM expressed high levels of both receptors (Figure 2B). 

Although we found very low or undetectable expression of C3aR and C5aR1 by either EMT6 or 4T1 cell lines, we investigated whether these cells might be capable of signal transduction in response to the dual C3aR/C5aR1 agonist, EP54. Although EP54 elicited a dose-dependent calcium response in J774 macrophages, it failed to mobilize intracellular calcium in either EMT6 or 4T1 cells (Figure 2C). Similarly, neither EP54 (10 µmol/L), recombinant mouse C3a (100 nmol/L) nor recombinant C5a (10 nmol/L) activated mitogen activated protein kinase (MAPK) signaling, as indicated by the inability to induce the phosphorylation of extracellular signal-regulated kinases (ERK) 1/2 (data not shown). 

### 3.3. Leukocyte Response to Pharmacological Modulation of C3aR/C5aR1 in Tumor-Bearing Mice 

Having found no evidence of receptor expression or signal activation by mammary carcinoma tumor cells, we next investigated whether EP54 may be acting on immune cells. Differential blood counts revealed that treatment with EP54 (1 mg/kg/day) resulted in a slight but significant increase in circulating leukocytes to 13.08 ± 0.42 × 10^6^ cells/mL compared with 12.19 ± 0.01 × 10^6^ cells/mL in vehicle-treated (control) mice (*p* < 0.05). There was no difference in neutrophil or monocyte numbers between groups, but lymphocytes were significantly increased in mice receiving EP54 (11.48 ± 0.51 × 10^6^ cells/mL) compared with vehicle-treated control mice (9.91 ± 0.581 × 10^6^ cells/mL; *p* < 0.05).

**Figure 3:**
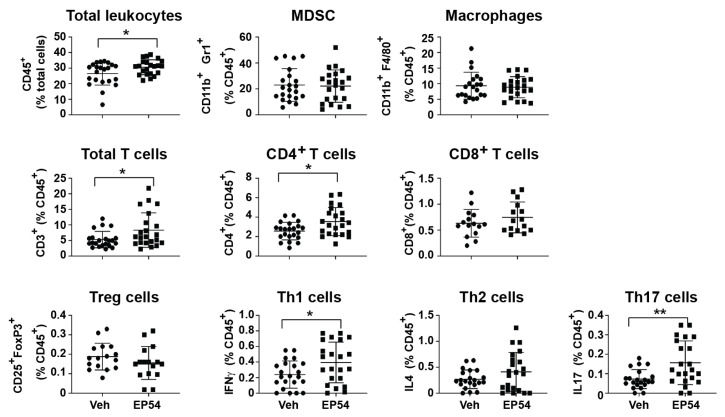
Effect of EP54-treatment on leukocyte sub-populations in mice with established 4T1 mammary tumors. Flow cytometric analysis of leukocyte sub-populations in tumor tissue from BALB/c mice treated with vehicle (control) ● or EP54 ■: total leukocytes (CD45^+^), myeloid derived suppressor cells (MDSC; CD11b^+^Gr-1^+^), macrophages (F4/80^+^), total (CD3^+^), CD4^+^ and CD8^+^ T lymphocytes; CD4^+^ T lymphocyte subsets: Treg (CD4^+^CD25^+^FoxP3^+^), Th1 (CD4^+^IFNγ^+^), Th2 (CD4^+^IL4^+^) and Th17 (CD4^+^IL17^+^). Results of three independent experiments (n = 6–8/group). Data expressed as % positive cells (mean ± SD); ** *p <* 0.01; * *p* < 0.05, Student’s *t*-test.

To further investigate the effect of EP54 on immune cells, we used the 4T1 model to analyze tumor infiltrating leukocyte populations by flow cytometry. As shown in Figure 3, there was a slight (but not significant) increase in total (CD45^+^) leukocytes infiltrating 4T1 tumors from EP54-treated mice. Although there were no differences in myeloid cell populations (myeloid derived suppressor cells (MDSC) or macrophages) between EP54- and control (vehicle)-treatment groups, the percentage of total (CD3^+^) T lymphocytes was significantly higher following EP54 treatment (8.3% ± 5.6%) compared with the control group (5.3% ± 2.6%; *p* < 0.05), as was the percentage of CD4^+^ T cells (3.6% ± 1.5% compared with 2.6% ± 0.9%; *p* < 0.05). Moreover, the proportions of CD4+ T cell subsets, Th1 and Th17, were increased within EP54-treated tumors. There was also a slight increase in the proportion of CD8^+^ T cells and reduction in Tregs, although these were not significant. Taken together, these results suggest that EP54 may inhibit mammary tumor growth by promoting an effective T cell-mediated response. 

## 4. Discussion

Chronic inflammation plays a critical role in the development and progression of cancer [47]. As key mediators of inflammation [48], the complement proteins C3a and C5a have been implicated for roles in tumorigenesis. Our laboratory and others have demonstrated that C3a and C5a promote tumor growth in a number of murine cancer models, including cervical [24], lung [25,30], ovarian [26], colon and melanoma [27,28]. However, the few studies investigating the role of these proteins in breast cancer models have produced conflicting results. Thus, the current study sought to clarify the role of complement proteins in murine syngeneic breast cancer models, EMT6 and 4T1. 

In accordance with the previous study by Vadrevu and co-workers, we found no effect of PMX53 on primary EMT6 mammary tumor growth [23]. However, treatment with a dual C3aR/C5aR1 agonist (EP54) inhibited development and growth of both EMT6 and 4T1 mammary tumors. These results are in agreement with the work of Kim et al., who demonstrated a protective role for C5a in the EMT6 mammary cancer model [21]. They are also in accordance with those of Bandini et al. [22], who showed that C3-deficiency (in which both C3a and C5a are lacking) accelerated tumor growth in a transgenic mouse model of mammary adenocarcinoma. This raises the question of whether the anti-tumor effects are mediated solely by C5a or whether C3a may also play a role. Our previous demonstration that C3aR signaling promotes 4T1 tumor growth, along with the evidence from Kim and co-workers that C5a alone has a protective role in EMT6 tumors, suggest that C5a is primarily responsible for the observed protective effects.

Another question still to be resolved is the mechanism by which EP54 exerts its anti-tumor effects. Kim and co-workers showed that C5a-expressing EMT6 tumors had high rates of apoptosis and cell cycle progression was blocked. Although this group demonstrated C5aR1 expression by EMT6 cells, we found that both C3aR and C5aR1 were undetectable on EMT6 and 4T1 cells at the protein level, suggesting that the anti-tumor effects of EP54 are indirect. Indeed, Kim and co-workers also showed that mice whose tumors regressed were immune to subsequent challenge with unmodified tumors (i.e., not expressing C5a), suggesting that at least some of the effects of C5a were indirect, via enhancement of the anti-tumor immune response. Moreover, Bandini et al. showed that mammary tumors from *C3*-deficient mice have a more immunosuppressive microenvironment [22]. The present study supports the premise that the tumor inhibitory effects of EP54 are due to immunoregulatory mechanisms, with EP54-treated mice showing increased tumor infiltration by T lymphocytes, in particular CD4+ T cell subsets, Th1 and Th17. While the presence of Th1 cells is linked to favorable prognoses in many cancers, the role of Th17 remains controversial [49]. However, Th17 cells have recently been identified as the most favorable prognostic indicator for triple-negative breast cancers with low T cell infiltrate [50]. Thus, the increased Th1–Th17 response in EP54-treated tumors suggests that these cells contribute to the anti-tumor response. Our results are in contrast to previous reports demonstrating that inhibition of C5aR1 signaling in MDSC favors Th1 and Th17 responses [23,51]. Although we found no significant difference in the proportions of tumor-infiltrating myeloid cells, qualitative differences are possible. For example, Markiewski and co-workers showed in the TC-1 cervical cancer model that C5a regulates the production of reactive oxygen and nitrogen species by MDSC [24]. In addition to immunoregulation, other effects are possible. For example, Nunez-Cruz and co-workers [26] demonstrated a primary role for C5a in neovascularization of ovarian cancers. Although beyond the scope of the present study, future studies could determine more precisely the mechanisms by which C3aR/C5aR1 agonism influences breast cancer growth, including potential effects on the function of myeloid and lymphocyte cell populations, and the ability to regulate tumor angiogenesis. 

The few studies investigating the role of complement proteins in mammary tumor models have yielded results that are in contrast to the majority of studies in other murine cancer models which show that pharmacological blockade of C5aR1 or C3aR inhibits tumor progression by limiting recruitment of immunosuppressive myeloid cells and Tregs into the tumor and promoting effective T cell responses [24,25,27,28,30]. The reasons for differing effects of complement proteins on different tumor types are yet to be determined, but likely include intrinsic differences in tumor mutational load, immunogenicity of tumor lines and immune profiles of the host mice [52]. One possible reason for differing responses between tumors is the variability in expression of complement receptors. Although we found no evidence of C3aR or C5aR1 expression by EMT6 or 4T1 mammary tumor cells, expression of these receptors has been reported for a range of human and mouse tumor cells, including melanoma [27], lung [53] and ovarian [31] cancers—all of which are inhibited by C3aR or C5aR1 receptor antagonism. While the lack of C3aR or C5aR1 expression by EMT6 and 4T1 tumor cells precludes direct effects of complement proteins on tumor growth, direct effects are possible for other (receptor-expressing) tumor models.

Compared with other common tumor models, 4T1 and EMT6 tumors are relatively immunogenic, with high levels of immune infiltration [54,55]. Our own experience shows that 4T1 mammary tumors have higher percentages of tumor-infiltrating leukocyte populations than poorly immunogenic B16.F0 melanoma tumors [28]. The site of tumor cell injection may also influence the response, due to tissue-specific variation in resident immune cells, differences in vascularization and the ability of immune cells to infiltrate the site [56]. The nature of the immune infiltrate is also likely to depend on the immune profile of the host strain. Most previous studies showing tumor-promoting effects of complement C3a and/or C5a have utilized tumor models syngeneic in C57Bl/6 mice whose immune system is skewed towards a Th1-M1 response [24,25,26,27,28,57]. Conversely, mammary tumor (EMT6 and 4T1 and the spontaneous neuT transgenic) models are on a BALB/c background in which Th2-M2 responses are dominant [58].

Differences in levels of complement proteins within the tumor microenvironment are also possible. Although C57Bl/6 and BALB/c mice have normal complement function [59], complement activation levels may differ, and tumor intrinsic complement production may also vary. As demonstrated in this study, both EMT6 and 4T1 cells express *C3* mRNA, and at levels that are relatively higher than other murine cell lines, such as B16 melanoma, MC38 colon carcinoma and Lewis lung carcinoma (LLC) [55]. As suggested by Gunn and co-workers [57], differing levels of tumor-derived complement proteins could contribute to observed differences in immune responses to the tumor, with low C5a levels inhibiting tumor growth by promoting Th1 cell differentiation, and high levels promoting Treg differentiation and stimulating tumor growth. Clearly, further work is required to clarify the roles of complement receptors in different tumor types, and how this is influenced by factors such as tumor immunogenicity, tumor site and the immune status of the host.

## 5. Conclusions

The present study demonstrated a protective role for C3aR/C5aR1 agonism in murine models of mammary carcinoma and suggests that this may be due to an enhanced T cell response. The results provide further evidence that complement proteins can exert distinct responses depending on the cancer type, possibly due to differences in the host’s immune response to the tumor. They also suggest that in the mammary tumor environment, exogenous C3a/C5a stimulation is required to trigger tumor inhibitory mechanisms. As proposed by Pio et al. [60], the immune system establishes a balance between tumor-promoting and tumor-inhibitory elements. By modulating the levels of complement activation, the balance may be altered towards a more (or less) favorable outcome. Thus, before complement-regulating drugs can be developed for clinical application, it is important to understand the mechanisms by which they exert their effects, and how this varies with cancer type.

## Supplementary Materials

The following are available online at https://www.mdpi.com/2073-4468/10/1/2/s1, Figure S1: Gating strategies for flow cytometric analysis of the tumor inflammatory infiltrate.

## References

[1] Bray F., Ferlay J., Soerjomataram I., Siegel R.L., Torre L.A., Jemal A. (2018). Global cancer statistics 2018: GLOBOCAN estimates of incidence and mortality worldwide for 36 cancers in 185 countries. CA Cancer J. Clin..

[2] Garrido-Castro A.C., Lin N.U., Polyak K. (2019). Insights into Molecular Classifications of Triple-Negative Breast Cancer: Improving Patient Selection for Treatment. Cancer Discov..

[3] Coussens L.M., Werb Z. (2002). Inflammation and cancer. Nature.

[4] Qu X., Tang Y., Hua S. (2018). Immunological Approaches towards Cancer and Inflammation: A Cross Talk. Front. Immunol..

[5] Reis E.S., Mastellos D.C., Ricklin D., Mantovani A., Lambris J.D. (2018). Complement in cancer: Untangling an intricate relationship. Nat. Rev. Immunol..

[6] Niculescu F., Rus H.G., Retegan M., Vlaicu R. (1992). Persistent complement activation on tumor cells in breast cancer. Am. J. Pathol..

[7] Chung L., Moore K., Phillips L., Boyle F.M., Marsh D.J., Baxter R.C. (2014). Novel serum protein biomarker panel revealed by mass spectrometry and its prognostic value in breast cancer. Breast Cancer Res..

[8] Imamura T., Yamamoto-Ibusuki M., Sueta A., Kubo T., Irie A., Kikuchi K., Kariu T., Iwase H. (2016). Influence of the C5a-C5a receptor system on breast cancer progression and patient prognosis. Breast Cancer.

[9] Ricklin D., Hajishengallis G., Yang K., Lambris J.D. (2010). Complement: A key system for immune surveillance and homeostasis. Nat. Immunol..

[10] Ostrand-Rosenberg S. (2008). Cancer and complement. Nat. Biotechnol..

[11] Gelderman K.A., Tomlinson S., Ross G.D., Gorter A. (2004). Complement function in mAb-mediated cancer immunotherapy. Trends Immunol..

[12] Geller A., Yan J. (2019). The Role of Membrane Bound Complement Regulatory Proteins in Tumor Development and Cancer Immunotherapy. Front. Immunol..

[13] Ouyang Q., Zhang L., Jiang Y., Ni X., Chen S., Ye F., Du Y., Huang L., Ding P., Wang N. (2016). The membrane complement regulatory protein CD59 promotes tumor growth and predicts poor prognosis in breast cancer. Int. J. Oncol..

[14] Maciejczyk A., Szelachowska J., Szynglarewicz B., Szulc R., Szulc A., Wysocka T., Jagoda E., Lage H., Surowiak P. (2011). CD46 Expression is an unfavorable prognostic factor in breast cancer cases. Appl. Immunohistochem. Mol. Morphol..

[15] Klos A., Wende E., Wareham K.J., Monk P.N. (2013). International Union of Pharmacology. LXXXVII. Complement peptide C5a, C4a, and C3a receptors. Pharmacol. Rev..

[16] Lo M.W., Woodruff T.M. (2020). Complement: Bridging the innate and adaptive immune systems in sterile inflammation. J. Leukoc. Biol..

[17] Li X.X., Lee J.D., Kemper C., Woodruff T.M. (2019). The complement receptor C5aR2: A powerful modulator of innate and adaptive immunity. J. Immunol..

[18] Pandey S., Maharana J., Li X.X., Woodruff T.M., Shukla A.K. (2020). Emerging Insights into the Structure and Function of Complement C5a Receptors. Trends Biochem. Sci..

[19] Wu M.C., Brennan F.H., Lynch J.P., Mantovani S., Phipps S., Wetsel R.A., Ruitenberg M.J., Taylor S.M., Woodruff T.M. (2013). The receptor for complement component C3a mediates protection from intestinal ischemia-reperfusion injuries by inhibiting neutrophil mobilization. Proc. Natl. Acad. Sci. USA.

[20] Coulthard L.G., Woodruff T.M. (2015). Is the complement activation product C3a a proinflammatory molecule? Re-evaluating the evidence and the myth. J. Immunol..

[21] Kim D.Y., Martin C.B., Lee S.N., Martin B.K. (2005). Expression of complement protein C5a in a murine mammary cancer model: Tumor regression by interference with the cell cycle. Cancer Immunol. Immunother..

[22] Bandini S., Curcio C., Macagno M., Quaglino E., Arigoni M., Lanzardo S., Hysi A., Barutello G., Consolino L., Longo D.L. (2013). Early onset and enhanced growth of autochthonous mammary carcinomas in C3-deficient Her2/neu transgenic mice. Oncoimmunology.

[23] Vadrevu S.K., Chintala N.K., Sharma S.K., Sharma P., Cleveland C., Riediger L., Manne S., Fairlie D.P., Gorczyca W., Almanza O. (2014). Complement c5a receptor facilitates cancer metastasis by altering T-cell responses in the metastatic niche. Cancer Res..

[24] Markiewski M.M., DeAngelis R.A., Benencia F., Ricklin-Lichtsteiner S.K., Koutoulaki A., Gerard C., Coukos G., Lambris J.D. (2008). Modulation of the antitumor immune response by complement. Nat. Immunol..

[25] Corrales L., Ajona D., Rafail S., Lasarte J.J., Riezu-Boj J.I., Lambris J.D., Rouzaut A., Pajares M.J., Montuenga L.M., Pio R. (2012). Anaphylatoxin C5a creates a favorable microenvironment for lung cancer progression. J. Immunol..

[26] Nunez-Cruz S., Gimotty P.A., Guerra M.W., Connolly D.C., Wu Y.Q., DeAngelis R.A., Lambris J.D., Coukos G., Scholler N. (2012). Genetic and pharmacologic inhibition of complement impairs endothelial cell function and ablates ovarian cancer neovascularization. Neoplasia.

[27] Nabizadeh J.A., Manthey H.D., Panagides N., Steyn F.J., Lee J.D., Li X.X., Akhir F.N.M., Chen W., Boyle G.M., Taylor S.M. (2019). C5a receptors C5aR1 and C5aR2 mediate opposing pathologies in a mouse model of melanoma. FASEB J..

[28] Nabizadeh J.A., Manthey H.D., Steyn F.J., Chen W., Widiapradja A., Md Akhir F.N., Boyle G.M., Taylor S.M., Woodruff T.M., Rolfe B.E. (2016). The Complement C3a Receptor Contributes to Melanoma Tumorigenesis by Inhibiting Neutrophil and CD4+ T Cell Responses. J. Immunol..

[29] Guglietta S., Chiavelli A., Zagato E., Krieg C., Gandini S., Ravenda P.S., Bazolli B., Lu B., Penna G., Rescigno M. (2016). Coagulation induced by C3aR-dependent NETosis drives protumorigenic neutrophils during small intestinal tumorigenesis. Nat. Commun..

[30] Kwak J.W., Laskowski J., Li H.Y., McSharry M.V., Sippel T.R., Bullock B.L., Johnson A.M., Poczobutt J.M., Neuwelt A.J., Malkoski S.P. (2018). Complement Activation via a C3a Receptor Pathway Alters CD4(+) T Lymphocytes and Mediates Lung Cancer Progression. Cancer Res..

[31] Cho M.S., Vasquez H.G., Rupaimoole R., Pradeep S., Wu S., Zand B., Han H.D., Rodriguez-Aguayo C., Bottsford-Miller J., Huang J. (2014). Autocrine effects of tumor-derived complement. Cell Rep..

[32] Boire A., Zou Y., Shieh J., Macalinao D.G., Pentsova E., Massague J. (2017). Complement Component 3 Adapts the Cerebrospinal Fluid for Leptomeningeal Metastasis. Cell.

[33] Rockwell S.C., Kallman R.F., Fajardo L.F. (1972). Characteristics of a serially transplanted mouse mammary tumor and its tissue-culture-adapted derivative. J. Natl. Cancer Inst..

[34] Pulaski B.A., Ostrand Rosenberg S. (2001). Mouse 4T1 Breast Tumor Model. Curr. Protoc. Immunol..

[35] Finch A.M., Wong A.K., Paczkowski N.J., Wadi S.K., Craik D.J., Fairlie D.P., Taylor S.M. (1999). Low-molecular-weight peptidic and cyclic antagonists of the receptor for the complement factor C5a. J. Med. Chem..

[36] Wu M.C.L., Lee J.D., Ruitenberg M.J., Woodruff T.M. (2020). Absence of the C5a Receptor C5aR2 Worsens Ischemic Tissue Injury by Increasing C5aR1-Mediated Neutrophil Infiltration. J. Immunol..

[37] Proctor L.M., Arumugam T.V., Shiels I., Reid R.C., Fairlie D.P., Taylor S.M. (2004). Comparative anti-inflammatory activities of antagonists to C3a and C5a receptors in a rat model of intestinal ischaemia/reperfusion injury. Br. J. Pharm..

[38] Manthey H.D., Thomas A.C., Shiels I.A., Zernecke A., Woodruff T.M., Rolfe B., Taylor S.M. (2011). Complement C5a inhibition reduces atherosclerosis in ApoE-/- mice. FASEB J..

[39] Campbell W.D., Lazoura E., Okada N., Okada H. (2002). Inactivation of C3a and C5a octapeptides by carboxypeptidase R and carboxypeptidase N. Microbiol. Immunol..

[40] Woodruff T.M., Strachan A.J., Sanderson S.D., Monk P.N., Wong A.K., Fairlie D.P., Taylor S.M. (2001). Species dependence for binding of small molecule agonist and antagonists to the C5a receptor on polymorphonuclear leukocytes. Inflammation.

[41] Finch A.M., Vogen S.M., Sherman S.A., Kirnarsky L., Taylor S.M., Sanderson S.D. (1997). Biologically active conformer of the effector region of human C5a and modulatory effects of N-terminal receptor binding determinants on activity. J. Med. Chem..

[42] Li X.X., Lee J.D., Massey N.L., Guan C., Robertson A.A.B., Clark R.J., Woodruff T.M. (2020). Pharmacological characterisation of small molecule C5aR1 inhibitors in human cells reveals biased activities for signalling and function. Biochem. Pharm..

[43] Kumar V., Lee J.D., Clark R.J., Noakes P.G., Taylor S.M., Woodruff T.M. (2020). Preclinical Pharmacokinetics of Complement C5a Receptor Antagonists PMX53 and PMX205 in Mice. ACS Omega.

[44] Hegde G.V., Meyers-Clark E., Joshi S.S., Sanderson S.D. (2008). A conformationally-biased, response-selective agonist of C5a acts as a molecular adjuvant by modulating antigen processing and presentation activities of human dendritic cells. Int. Immunopharmacol..

[45] Tomayko M.M., Reynolds C.P. (1989). Determination of subcutaneous tumor size in athymic (nude) mice. Cancer Chemother. Pharm..

[46] Spang-Thomsen M., Nielsen A., Visfeldt J. (1980). Growth curves of three human malignant tumors transplanted to nude mice. Exp. Cell Biol..

[47] Greten F.R., Grivennikov S.I. (2019). Inflammation and Cancer: Triggers, Mechanisms, and Consequences. Immunity.

[48] Klos A., Tenner A.J., Johswich K.O., Ager R.R., Reis E.S., Kohl J. (2009). The role of the anaphylatoxins in health and disease. Mol. Immunol..

[49] Bailey S.R., Nelson M.H., Himes R.A., Li Z., Mehrotra S., Paulos C.M. (2014). Th17 cells in cancer: The ultimate identity crisis. Front. Immunol..

[50] Faucheux L., Grandclaudon M., Perrot-Dockes M., Sirven P., Berger F., Hamy A.S., Fourchotte V., Vincent-Salomon A., Mechta-Grigoriou F., Reyal F. (2019). A multivariate Th17 metagene for prognostic stratification in T cell non-inflamed triple negative breast cancer. Oncoimmunology.

[51] Markiewski M.M., Vadrevu S.K., Sharma S.K., Chintala N.K., Ghouse S., Cho J.H., Fairlie D.P., Paterson Y., Astrinidis A., Karbowniczek M. (2017). The Ribosomal Protein S19 Suppresses Antitumor Immune Responses via the Complement C5a Receptor 1. J. Immunol..

[52] Yu J.W., Bhattacharya S., Yanamandra N., Kilian D., Shi H., Yadavilli S., Katlinskaya Y., Kaczynski H., Conner M., Benson W. (2018). Tumor-immune profiling of murine syngeneic tumor models as a framework to guide mechanistic studies and predict therapy response in distinct tumor microenvironments. PLoS ONE.

[53] Ajona D., Zandueta C., Corrales L., Moreno H., Pajares M.J., Ortiz-Espinosa S., Martinez-Terroba E., Perurena N., de Miguel F.J., Jantus-Lewintre E. (2018). Blockade of the Complement C5a/C5aR1 Axis Impairs Lung Cancer Bone Metastasis by CXCL16-mediated Effects. Am. J. Respir. Crit. Care Med..

[54] Mosely S.I., Prime J.E., Sainson R.C., Koopmann J.O., Wang D.Y., Greenawalt D.M., Ahdesmaki M.J., Leyland R., Mullins S., Pacelli L. (2017). Rational Selection of Syngeneic Preclinical Tumor Models for Immunotherapeutic Drug Discovery. Cancer Immunol. Res..

[55] Zhong W., Myers J.S., Wang F., Wang K., Lucas J., Rosfjord E., Lucas J., Hooper A.T., Yang S., Lemon L.A. (2020). Comparison of the molecular and cellular phenotypes of common mouse syngeneic models with human tumors. BMC Genom..

[56] Devaud C., Westwood J.A., John L.B., Flynn J.K., Paquet-Fifield S., Duong C.P., Yong C.S., Pegram H.J., Stacker S.A., Achen M.G. (2014). Tissues in different anatomical sites can sculpt and vary the tumor microenvironment to affect responses to therapy. Mol. Ther. J. Am. Soc. Gene Ther..

[57] Gunn L., Ding C., Liu M., Ma Y., Qi C., Cai Y., Hu X., Aggarwal D., Zhang H.G., Yan J. (2012). Opposing roles for complement component C5a in tumor progression and the tumor microenvironment. J. Immunol..

[58] Mills C.D., Kincaid K., Alt J.M., Heilman M.J., Hill A.M. (2000). M-1/M-2 macrophages and the Th1/Th2 paradigm. J. Immunol..

[59] Sellers R.S., Clifford C.B., Treuting P.M., Brayton C. (2012). Immunological variation between inbred laboratory mouse strains: Points to consider in phenotyping genetically immunomodified mice. Vet. Pathol..

[60] Pio R., Ajona D., Lambris J.D. (2013). Complement inhibition in cancer therapy. Semin. Immunol..

