# Supplementary material for: An Immunoregulatory Role for Complement Receptors in Murine Models of Breast Cancer
Source: Antibodies (Basel). 2021 Jan 8;10(1):2. doi: 10.3390/antib10010002 (PMC7838807; doi:10.3390/antib10010002)
Supplement: Supplementary file 1 [file antibodies-10-00002-s001.pdf]

Akhir Supplemental Data

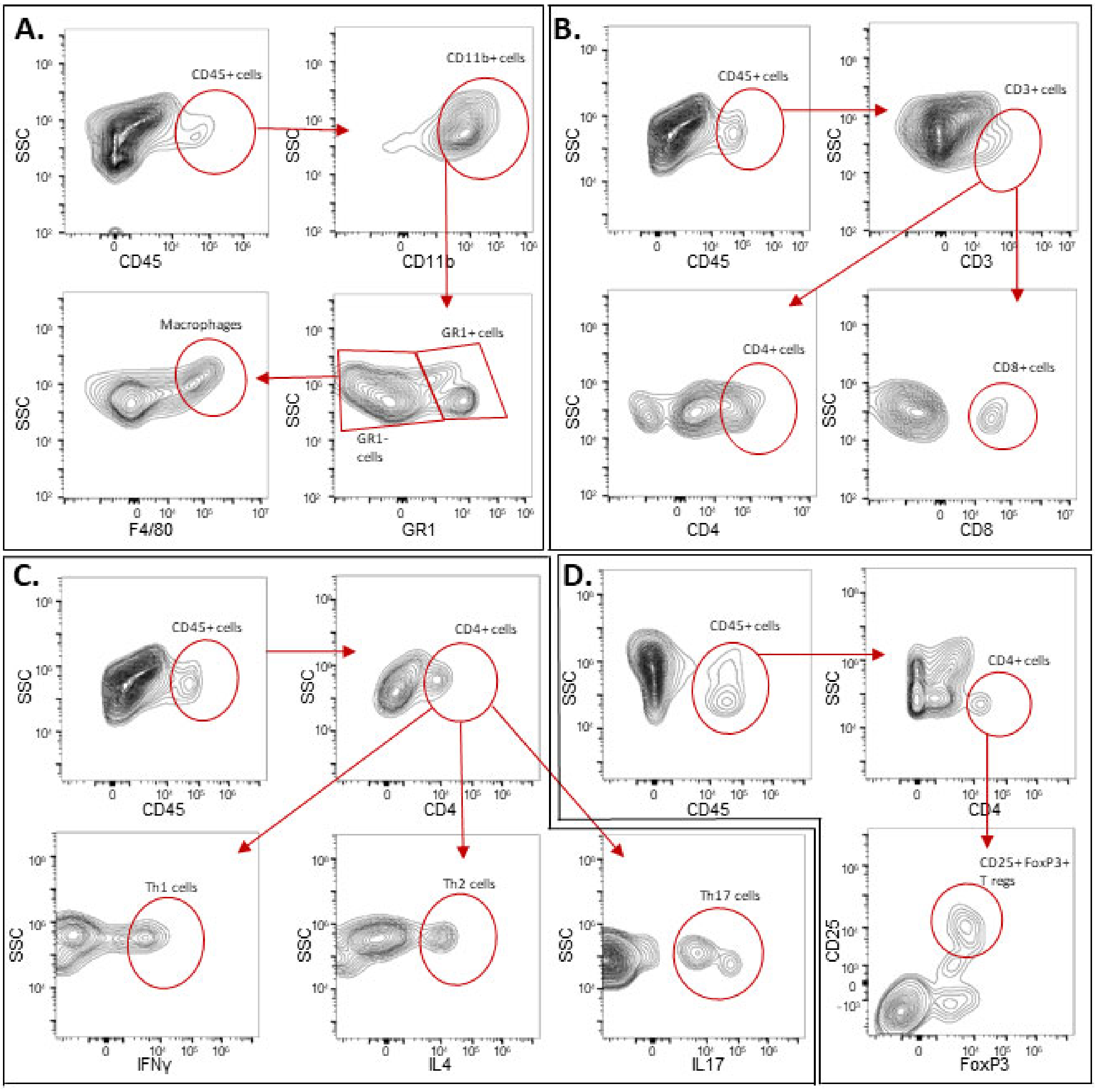

Supplemental Figure 1. Gating strategies for flow cytometric analysis of the tumor inflammatory infiltrate. Cells were first gated on viability and size (SSC-A vs. FSC-A; not shown), then $CD45^+$ cells (total leukocytes) were (***A***) gated for $CD11b^+$ (myeloid cells) which were sub-gated for $Gr\text{-}1^+$ cells, with $F480^+$ (macrophages) derived from the $Gr\text{-}1^-$ population; (***B***) $CD45^+$ cells were also gated for total T lymphocytes ($CD3^+$) which were further separated into $CD8^+$ and $CD4^+$ sub-sets. For populations with intracellular cytokines, $CD4^+$ cells were gated (***C***) into Th1 ($CD4^+IFN\gamma^+$), Th2 ($CD4^+IL4^+$) Th17 ($CD4^+IL17^+$) and (***D***) Tregs ($CD4^+CD25^+FOXP3^+$). For each gated population, the frequency of cells was expressed as percentage of $CD45^+$ or $CD4^+$ cells.
